# The timing of oesophageal dilatations in anastomotic stenosis after one-stage anastomosis for congenital oesophageal atresia

**DOI:** 10.1186/s13019-021-01656-y

**Published:** 2021-10-09

**Authors:** Xue-Jie Gao, Jin-Xi Huang, Qiang Chen, Song-Ming Hong, Jun-Jie Hong, Hong Ye

**Affiliations:** 1 Department of Pediatrics, Fujian Maternity and Child Health Hospital, Affiliated Hospital of Fujian Medical University, Fuzhou, China; 2 Department of Cardiothoracic Surgery, Fujian Branch of Shanghai Children’s Medical Center, Fuzhou, China; 3 Department of Pediatrics, Fujian Branch of Shanghai Children’s Medical Center, Fuzhou, China

**Keywords:** Congenital oesophageal atresia, Anastomotic stenosis, Timing of oesophageal dilatation, Anastomotic fistula, Refractory anastomotic stenosis

## Abstract

**Background:**

In infants with congenital oesophageal atresia, anastomotic stenosis easily occurs after one-stage oesophageal anastomosis, leading to dysphagia. In severe cases, oesophageal dilatation is required. In this paper, the timing of oesophageal dilatation in infants with anastomotic stenosis was investigated through retrospective data analysis.

**Methods:**

The clinical data of 107 infants with oesophageal atresia who underwent one-stage anastomosis in our hospital from January 2015 to December 2018 were retrospectively analysed. Data such as the timing and frequency of oesophageal dilatation under gastroscopy after surgery were collected to analyse the timing of oesophageal dilatation in infants with different risk factors.

**Results:**

For infants with refractory stenosis, the average number of dilatations in the early dilatation group (the first dilatation was performed within 6 months after the surgery) was 5.75 ± 0.5, which was higher than the average of 7.40 ± 1.35 times in the normal dilatation group (the first dilatation was performed 6 months after the surgery), P = 0.038. For the infants with anastomotic fistula and anastomotic stenosis, the number of oesophageal dilatations in the early dilatation group was 2.58 ± 2.02 times, which was less than the 6.38 ± 2.06 times in the normal dilatation group, P = 0.001. For infants with non-anastomotic fistula stenosis, early oesophageal dilatation could not reduce the total number of oesophageal dilatations.

**Conclusion:**

Starting to perform oesophageal dilatation within 6 months after one-stage anastomosis for congenital oesophageal atresia can reduce the required number of dilatations in infants with postoperative anastomotic fistula and refractory anastomotic stenosis.

## Background

Congenital oesophageal atresia is a rare malformation of the digestive system, with an average of 1 in every 2500–4000 newborns suffering from oesophageal atresia (EA) [1]. Approximately 50% of infants may have associated congenital malformations in other systems [2]. Progress has been made in the treatment of EA in the past 20 years, and the success rate of treatment has gradually increased to over 90%. Thoracoscopic surgery has gradually become the main method of treatment for EA. However, anastomotic stenosis is still the most common postoperative complication (17–59%) that often occurs in the first year after surgery [3, 4]. It may lead to recurrent respiratory complications and malnutrition [5]. Thoracoscopic surgery and anastomotic fistula may be closely related to the occurrence of anastomotic stenosis, and severe anastomotic fistula may even lead to refractory anastomotic stenosis [6].

Anastomotic stenosis is defined as postoperative feeding difficulties or narrowness confirmed by gastrointestinal radiography and endoscopy [7]. Severe anastomotic stenosis is one of the main reasons for poor postoperative quality of life in infants [8]. At present, the main treatment for oesophageal stenosis is regular oesophageal dilatation [9], including endoscopic oesophageal probe dilatation and balloon dilatation, and some infants with severe stenosis need to undergo resection of stenotic segments and oesophageal end-to-end anastomosis or even oesophageal replacement therapy [10, 11]. Refractory stenosis is defined as severe dysphagia requiring at least five oesophageal dilatations, and the interval between adjacent dilatations cannot exceed 4 weeks according to the European Nutritional Guidelines for Paediatric Gastroenterology published by the European Society of Gastroenterology [12].

Through retrospective analysis, we hope to find the appropriate time for dilatation in infants with refractory stenosis.

## Methods

Data from a total of 107 infants with EA who underwent one-stage anastomosis in our hospital from January 2015 to December 2018 were retrospectively analysed. The data collected included birth weight, gestational week, surgical method, other systemic malformations, and anastomotic fistula (Table 1). All patients included in this study had varying degrees of dysphagia and had esophageal stenosis confirmed by esophagography. All esophageal dilatation procedures were performed under endotracheal intubation and general anesthesia, followed by routine use of antibiotics to prevent infection and hemostatic drugs. The accurate measurement of the distance of esophageal defect is the end-to-end distance of the esophagus after the esophageal bed is dissociated during the operation. Esophageal dilatation procedure: we evaluate the size of the anastomosis during digestive endoscopy, select an appropriate probe for the first dilatation, for the second dilatation probe, we chose a probe 2 mm larger than the first one, and an additional 2 mm larger probe is used for the third expansion.

**Table 1:** General information

Sex (male/female)	74/33
Gestational age (week)	38.51 ± 1.61
Birth weight (kg)	2.83 ± 0.48
Surgical method (endoscopy/open)	49/58
Other associated systemic malformations (yes/no)	33/74
Cardiovascular system malformation	14
Malformation of the digestive system	8
Urological deformity	4
Skeletal system malformation	11
Respiratory malformation	9
Other malformation	5
Length of defect (cm)	1.53 ± 0.96
Anastomotic fistula	28
Anastomotic stricture	56
Number of oesophageal dilatations	3.5 ± 2.4
Refractory stenosis (continuous dilatation ≥ 5)	14

Data related to oesophageal dilatation in all infants were collected and analysed, including the infants' age at the time of first oesophageal dilatation and the total number of oesophageal dilatations. Postoperative anastomotic stenosis was observed in 56 infants, and oesophageal dilatation was performed in 48 infants.

SPSS 2.0 software was used to analyse the data, and t tests were used for the oesophageal dilatation analysis. P < 0.05 was considered statistically significant.

## Results

**Fig. 1:**
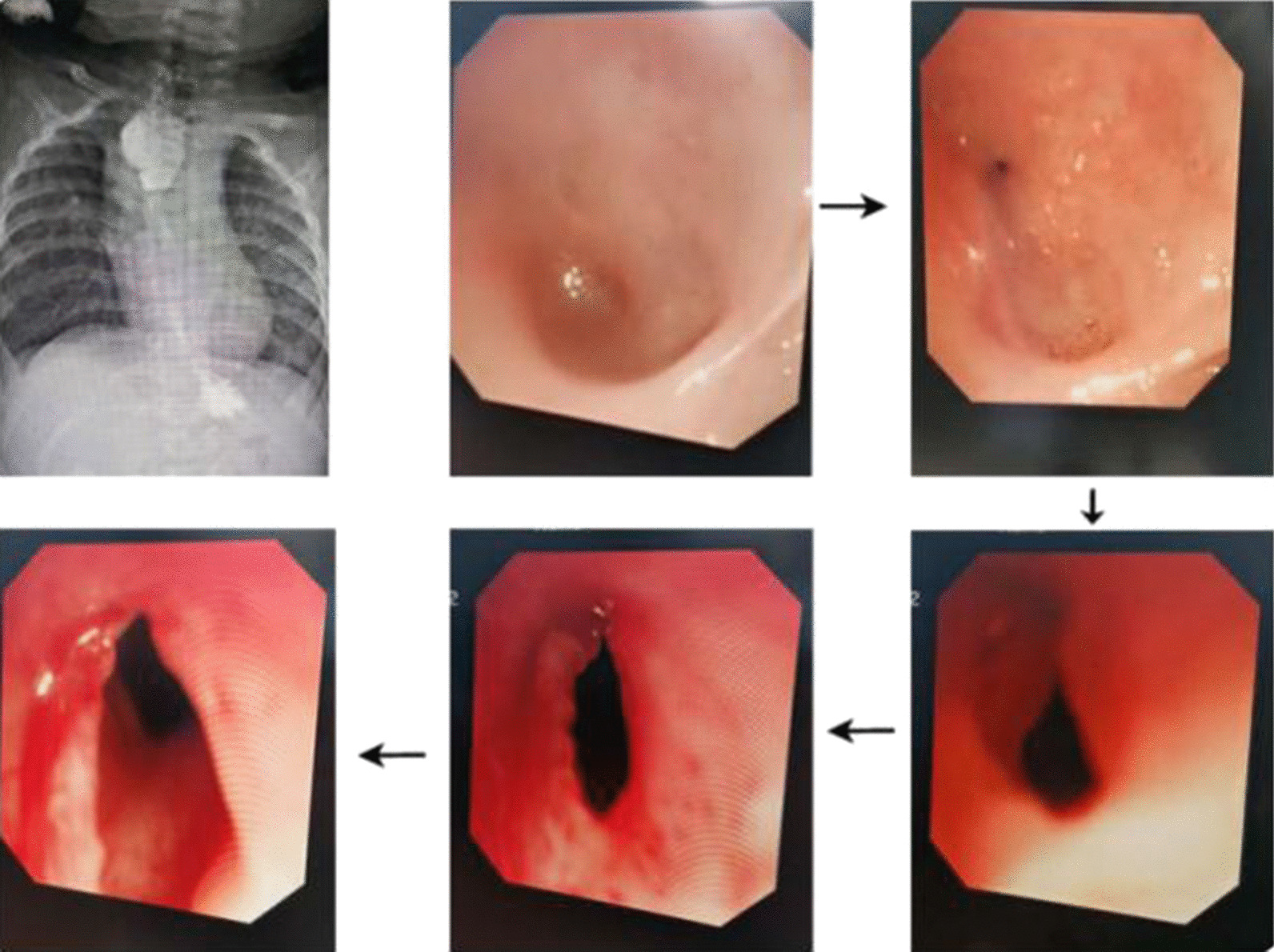
Refractory anastomotic stenosis after anastomotic fistula

1. Refractory stenosis: There is no definition of "early" or "normal" dilatation timing. Dai et al.'s study[13] divided dilatation timing into early dilatation (the first dilatation was less than or equal to 6 months after the surgery) and normal dilatation (the first dilatation was more than 6 months after the surgery). In our study, for infants with refractory stenosis, the average number of dilatations in the early dilatation group was 5.75 ± 0.5 compared with 7.40 ± 1.35 in the normal dilatation group, P = 0.038. For infants without refractory stenosis, there was no statistically significant difference in the average number of dilatations between the two groups (Table 2).
2. Stenosis occurring after anastomotic fistula: We analysed the number of dilatations in the anastomotic fistula group and non-anastomotic fistula group, and the results indicated that for infants with anastomotic fistula, the average number of dilatations was 2.58 ± 2.02 in the early dilatation group and 6.38 ± 2.06 in the normal dilatation group (P < 0.05). For infants without anastomotic fistula, there was no significant difference in the number of dilatations between the early and normal dilatation groups (Table 3). Early oesophageal dilatation is considered helpful for infants with oesophageal stenosis after anastomotic fistula.
3. Anastomotic stenosis occurring after anastomotic fistula appeared more serious and even became a pinpoint anastomoses (Fig. 1). This child had the most dilatations, and severe anastomotic stenosis appeared one month after the surgery, which was then cured after conservative treatment. Through continuous dilatation and long-term indwelling of the gastric tube, a probe with a diameter of 11 mm was gradually allowed to pass the anastomoses, and the dysphagia of this child was gradually relieved during the dilatation process. This child received dilatation once every two weeks, for a total of 11 times.

**Table 2:** The average number of dilatations

	Early dilatation group	Normal dilatation group	t	P
Refractory stenosis	5.75 ± 0.5	7.40 ± 1.35	2.33	0.038
Non-refractory stenosis	2.05 ± 0.97	2.13 ± 0.92	0.247	0.807

**Table 3:** The average number of dilatations

	Early dilatation group	Normal dilatation group	t	P
With anastomotic fistula	2.58 ± 2.02	6.38 ± 2.06	4.857	0.001
Without anastomotic fistula	1.58 ± 0.52	2.23 ± 1.59	1.346	0.192

## Discussion

1. The treatment of oesophageal stenosis still remains a problem, and oesophageal dilatation has a positive effect on oesophageal stenosis [14]. Lang et al. found that oesophageal balloon dilatation had the same effect as probe dilatation [15]. Studies have calculated that the incidence of oesophageal perforation after probe dilatation is 0.9% [16] and after balloon dilatation is 1.5%[17]. Esophageal dilation should not be increased by more than 3 mm in a single endoscopy to reduce the risk of perforation, the so-called "rule of 3"[18, 19]. Clark, SJ founded that balloon dilations that expanded the initial esophageal osis ≤ 5 mm in a pediatric population appear to not unduly increase the risk of perforation in his research[20]. Non-adherence to the "rule of 3" does not appear to increase the risk of adverse events, particularly perforation, after esophageal dilation using bougie dilators[21]. All the infants in our group received probe dilatation. Three successive progressive probe dilatations were performed in a esophageal dilatation operation. The diameter of the first probe was determined by the size of the anastomosis. The diameter of the second probe was 2 mm larger than that of the first, and the diameter of the third probe was 4 mm larger than that of the first. We think this is a safe and reliable method of probe selection and esophageal dilation.
2. Esophageal dilatation is generally carried out after the addition of complementary food in infants, and some infants have to carry out esophageal dilatation earlier because of feeding difficulties. In infants with refractory stenosis, severe anastomotic scar hyperplasia resulted in smaller anastomotic diameter and more dilating tension. Premature anastomotic dilatation may lead to anastomotic perforation [22]. Debourdeau[23] reported a long-term result of repeated and sustained esophageal dilations in patients with refractory strictures. They found that the planned expansion group required significantly fewer expansions than the on-demand expansion group, scheduled expansions were associated with a higher probability of final success and a shorter treatment duration.
  Therefore, although early dilatation is necessary for some infants due to severe feeding difficulties, we also recommend that it should be performed 3–6 months after surgery. In some cases, we made an empirical judgment during the first dilation, such as small anastomosis, high probe resistance during dilation, and severe elastic retraction during subsequent dilation. For children with these conditions, choosing early dilation can help reduce the number of dilations. Anastomotic scar hyperplasia usually occurs 3–4 months after surgery. For refractory anastomotic stricture, dilatation within 3–6 months may help to relieve the narrowing caused by scarring. This is our experience in the treatment process. Data analysis also confirmed that early dilatation chosen for infants with refractory stenosis can reduce the number of dilatations.
3. The dysphagia caused by non-refractory stenosis is relatively light, and satisfactory results can often be obtained only after 1–2 dilatations [24]. Our recommendation is that for refractory stenosis, early dilatation and shortening of the dilatation interval may reduce the required number of dilatations, while for infants with non-refractory stenosis, it may be safer to perform dilatation 6 months after surgery.

For infants with unsatisfactory effects of continuous dilatation, other conservative treatment methods have also been reported, including sterol injection, oesophageal stent placement and endoscopic stenosis incision [7, 25]. When conservative treatment fails, oesophageal replacement therapy may eventually be required [2].

The limitation is that this study is a retrospective study. The symptoms were subjectively assessed by the parents of the infants, and the anastomosis was further assessed by oesophagography, which may have some errors caused by subjective differences. We hope to design relevant prospective studies to further confirm the accuracy of the conclusions.

## Conclusion

Esophageal dilation started within 6 months after primary anastomosis of EA can reduce the number of dilations required for infants with refractory anastomotic stenosis.

## Data Availability

Written informed consent was obtained from the parents of the patients for publication of this article.

## Abbreviation

EA: Oesophageal atresia
